# Examining the impact of the COVID-19 pandemic on hospital-associated *Clostridioides difficile* infection

**DOI:** 10.1017/ash.2023.388

**Published:** 2023-09-29

**Authors:** Michael Ray, Jon Furuno, Luke Strnad, Eric Lofgren, Jessina McGregor

## Abstract

**Background:** The epidemiology of *Clostridioides difficile* infection (CDI) is complex, and the COVID-19 pandemic has had extreme impacts on known risk factors such as comorbidity burden and antibiotic prescribing. However, whether these changes have affected the incidence of hospital-associated CDI (HA-CDI) remains unknown. We compared incidence and trends of HA-CDI before and after the pandemic onset, and we assessed the impact of changes in key CDI-related risk factors. **Methods:** We conducted an interrupted time-series study (March 2018–March 2021) of adult inpatients hospitalized 4 or more days with no known CDI on admission at a 576-bed academic medical center. Our primary outcome was monthly HA-CDI per 10,000 patient days. We performed segmented linear regression to compare the preinterruption trend in HA-CDI rate to the postinterruption slope and level change. We established a series of 30-day intervals before and after the interruption timepoint of March 23, 2020, which corresponds with the Oregon stay-at-home executive order. The data included 24 preinterruption time points and 12 postinterruption time points. We also assessed changes in slope and trend for known HA-CDI risk factors. **Results:** We included 34,592 inpatient encounters in our prepandemic period and 10,932 encounters in our postinterruption period. The mean prepandemic HA-CDI rate was 4.07 cases per 10,000 patient days. After the pandemic onset, the rate was 3.6 per 10,000 patient days. However, the observed differences in rate (both in terms of slope and level) were not statistically significant (*P* = .90 for level; *P* = .60 for slope change). We observed a significant decrease in admissions per 30 days (1,441 vs 911; level-change *P* < .0001) and a slight increase in the mean number of Elixhauser comorbidities (1.96 vs 2.07; level-change *P* = .05). We also observed significant increases in both frequency and intensity of antibiotic use, with an increase average days of therapy per encounter (5.8 vs 7.2; level-change *P* = .01; slope-change *P* < .0001) and in antibiotic spectrum index (ASI) points per antibiotic day (4.4 vs 4.9; *P* < .0001). We observed a consistent downward trend for case days of CDI colonization pressure per hospital day (preinterruption slope *P* < .0001), which remained consistent after the pandemic onset (*P* = 0.5 for postinterruption slope change) (Fig. 1). **Conclusions:** Despite significant increases in high-intensity antibiotic use and comorbidity burden, we did not observe significant differences in HA-CDI after the pandemic onset. This may be due to the significant decrease in colonization pressure in the postpandemic period. Further research is required to fully understand the impact of the pandemic-related changes on

**Figure:**
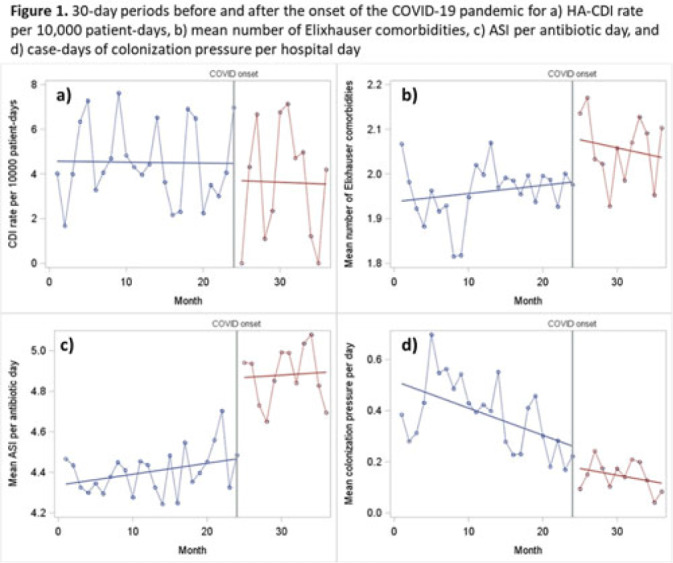

**Disclosures:** None

